# Concrete with Partial Substitution of Waste Glass and Recycled Concrete Aggregate

**DOI:** 10.3390/ma15020430

**Published:** 2022-01-07

**Authors:** Jawad Ahmad, Rebeca Martínez-García, Jesús de-Prado-Gil, Kashif Irshad, Mohammed A. El-Shorbagy, Roman Fediuk, Nikolai Ivanovich Vatin

**Affiliations:** 1 Department of Civil Engineering, Military College of Engineering (Nust), Risalpur 24080, Pakistan; 2 Department of Mining Technology, Topography, and Structures, University of León, Campus de Vegazana s/n, 24071 León, Spain; rmartg@unileon.es (R.M.-G.); jesusdepradogil@gmail.com (J.d.-P.-G.); 3 Interdisciplinary Research Center for Renewable Energy and Power Systems (IRC-REPS), King Fahd University of Petroleum & Minerals, Dhahran 31261, Saudi Arabia; Kashif.irshad@kfupm.edu.sa; 4 K.A. CARE Energy Research & Innovation Center at Dhahran, Dhahran 31261, Saudi Arabia; 5 Department of Mathematics, College of Science and Humanities in Al-Kharj, Prince Sattam bin Abdulaziz University, Al-Kharj 11942, Saudi Arabia; mohammed_shorbagy@yahoo.com; 6 Polytechnic Institute, Far Eastern Federal University, 690922 Vladivostok, Russia; fedyuk.rs@dvfu.ru; 7 Peter the Great Saint Petersburg Polytechnic University, 195251 Saint Petersburg, Russia; vatin@mail.ru

**Keywords:** sustainable concrete, compressive strength, split tensile strength, waste glass

## Abstract

The current practice of concrete is thought to be unsuitable because it consumes large amounts of cement, sand, and aggregate, which causes depletion of natural resources. In this study, a step towards sustainable concrete was made by utilizing recycled concrete aggregate (RCA) as a coarse aggregate. However, researchers show that RCA causes a decrease in the performance of concrete due to porous nature. In this study, waste glass (WG) was used as a filler material that filled the voids between RCA to offset its negative impact on concrete performance. The substitution ratio of WG was 10, 20, or 30% by weight of cement, and RCA was 20, 40, and 60% by weight of coarse aggregate. The slump cone test was used to assess the fresh property, while compressive, split tensile, and punching strength were used to assess the mechanical performance. Test results indicated that the workability of concrete decreased with substitution of WG and RCA while mechanical performance improved up to a certain limit and then decreased due to lack of workability. Furthermore, a statical tool response surface methodology was used to predict various strength properties and optimization of RCA and WG.

## 1. Introduction

Environmental groups are pushing for the use of RCA and solid wastes such as slag, fly ash, and silica fume in construction projects. The global demand for aggregates for construction is expected to reach 47 billion metric tons per year in 2023 [1]. The solution is to reuse or include solid waste byproducts including fly ash, silica fume, bottom ash, slag, and glass waste into the concrete manufacturing process [1,2,3,4]. These concrete advances diminish the negative impacts of the concrete industry in affordable and natural issues by providing low costs, high strength properties, and ecological amicability [5,6,7]. Currently, the usage of reused aggregate concrete, which is made from waste concrete brought in from building sites and demolition, is being promoted to help alleviate the global scarcity of regular aggregate. Furthermore, by recycling waste concrete aggregate, concerns associated with removing the massive volume of waste concrete generated on building sites and demolition activities might be reduced up to certain extent [1].

By volume, RA is made up of 60–70 percent natural aggregates and 30–40 percent aged cement mortar. Parent concrete qualities, workability, mix percentage, and other factors influence the compressive strength and other properties of recyled aggregate concrete [8,9,10]. When compared to concrete with natural aggregates, the mechanical and durability performance of RCA is worse. By using fractional substitution of pozzolanic components and mineral admixtures, RCA’s inferior performance might be improved. By filling the RCA porosity microstructure and lowering RCA permeability, these compounds and admixtures improve durability [11,12]. Using RCA as coarse aggregate in concrete has a number of environmental advantages. However, because of its porous nature, RCA has been shown to reduce the mechanical performance of concrete. As a result, some filler material must be added to RCA, filling the gaps and resulting in a more compact mass. There are various filler materials, such as fly ash [13], silica fume [14,15], waste glass [16], wheat straw ash [4], marble waste [17], bentonite clay [18], as well as GGBS [19]. In this study, waste glass was used as the filler material.

As of 2005, the overall worldwide waste glass manufacture valuation was 130 Mt, in which the European Union, China, and the USA manufactured roughly 33 Mt, 32 Mt, and 20 Mt, respectively [20]. As glass is nonrecyclable, glass-dumping in a landfill creates an environmental problem. Because of these reasons, the use of waste glass and waste marble materials from industries came into the picture to reduce the waste from manufacturing units and decrease the scarcity of basic natural resources.

On the other hand, the yearly worldwide cement manufacture has moved 2.8 billion tons and is supposed to rise to some four billion tons each year. Cement production is challenging because of expense rises in energy resources, decreasing CO_2_ releases, and depletion of natural resources [9,21,22]. Several industrial byproducts have been used effectively in concrete, including silica fume, ground granulated blast furnace slag, and fly ash [23,24]. The practice of waste glass to change cement could decrease the cost of concrete and the depletion of cement, thereby precisely decreasing the CO_2_ production associated with cement manufacture.

Glass exhibits pozzolanic behavior if the particle size is smaller than 75 µm. Silica in glass interacts with calcium hydrates (Ca(OH)_2_) to form calcium silicate hydrate (CSH) [25]. Glass has also been found to exhibit pozzolanic properties when the particle size is smaller than 150 µm. However, the pozzolanic behaviors increase as particle size decreases, i.e., 35 µm shows better results than 150 µm [26,27].

It has been concluded that slump is decreased with the addition of waste glass. However, compressive and flexure strength increases up to 20% replacement and then decreases gradually [28]. It has been concluded that the flexure strength of the beam is increased with the addition of waste glass [29]. It has been shown that the compression strength level was 13.64% at seven days and 2.18% at 28 days at 20% replacement of glass [30]. Finely ground glass powder has been found to exhibit pozzolanic properties which increase the strength of concrete. [31,32]. The results show that compressive strength is about 12%, 2.5%, and 1.5% higher than that of reference concrete with substitution of waste glass. However, a high dosage of waste glass may negatively impact the strength property of concrete due to dilution effects. Excessive silica without the necessary calcium (CH) forms weak pockets (the alkali-silica reaction), and as a result, strength starts to decrease [25]. Therefore, it is essential to choose the optimum dose of waste glass for the better mechanical performance of concrete.

The literature shows that RCA decreases the performance of concrete due to its porous nature, and waste glass has the credibility to be used as a filler material. Therefore, waste glass was used as a filler material that filled the voids of RCA and offset its negative impact on the mechanical performance of concrete. Furthermore, a response surface methodology was used to optimize RCA and WG. The overall study demonstrates that the combined substitution of WG and RCA successfully improved mechanical performance considerably.

## 2. Materials and Methods

### 2.1. Materials

#### 2.1.1. Cement

As per ASTM C150 Type-1, Ordinary Portland cement (Bestway cement Islamabad Pakistan) was used throughout the experimental work to prepare the mix for all batches. The physical and chemical aspects of cement used in this study are shown in Table 1.

#### 2.1.2. Waste Glass

**Figure 1:**
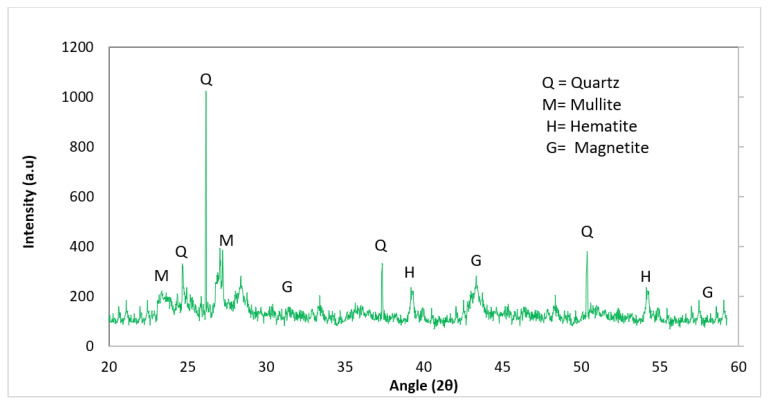
XRD result of waste glass.

Waste glass was procured from a local shop Kohat Pakistan. The waste glass used in the research was transparent soda-lime glass. The waste glass was ground using ball mill grinding equipment at the PCSIR lab in Peshawar, Pakistan, to reach a particle size equivalent to or less than that of the cement particle size. The physical and chemical aspects of waste glass used in this study are shown in Table 2, while X-ray diffraction (XRD) is given in Figure 1. At 26, 38, and 50 degrees, many amorphous peaks of quartz (Q) in the XRD analysis of WG showed the major amorphous nature of WG. The chemical composition of WG showed that the material may have had pozzolanic potential, which can be attributed to the accumulative chemical composition of SiO_2_, Al_2_O_3_, and Fe_2_O_3_ exceeding 70%.

#### 2.1.3. Aggregates

**Figure 2:**
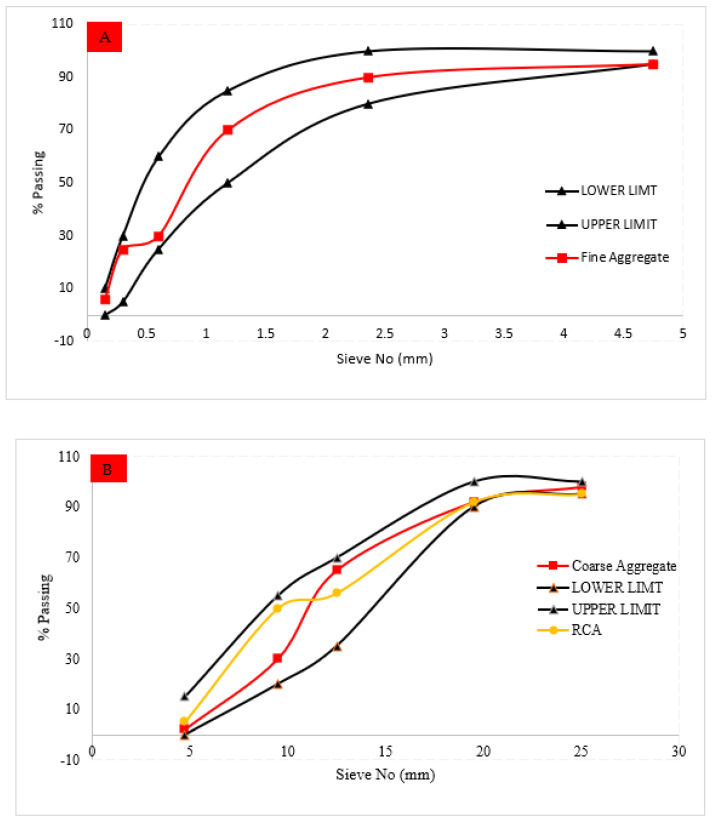
Particle size distribution curve of (**A**) fine aggregate and (**B**) coarse aggregate.

In saturated surface dry conditions, natural river sand was employed as a fine aggregate in all batches. For all batches, normal weight coarse aggregate with a maximum size of 25 mm was employed in saturated surface dry conditions. RCA of 25 mm maximum size was obtained by crushing waste concrete. A variety of tests were carried out on the aggregates to assess its physical properties, and the results are given in Table 3. For aggregate gradation (sieve analysis), an ASTM standard (ASTM33/C33M-13) was utilized, as indicated in Figure 2.

### 2.2. Response Surface Methodology (RSM)

RSM is a mathematical and statistical combined technique to estimate relations between a set of input independent variables and output variables [33]. Experimental results are required, and then based on these experimental results, a numerical model is developed [34]. Additionally, RSM is additionally used as an experimental design technique for the modeling and analysis of difficulties in which a response of interest is assessed by several variables [33,35]. This method is generally used when several input variables affect the output variable. Equation (1) (first-order model) was utilized to calculate the quantity of the experimentation [36].

(1) $$Y=\beta 0+\overset{k}{\underset{i=1}{\sum }}\beta iX_{i}+\varepsilon$$

Equation (2) (second-order model) illustrated the model utilized to estimate the responses and define the relationship between the independent variables.

(2) $$Y=\beta 0+\overset{k}{\underset{i=1}{\sum }}\beta \mathrm{ii}X_{i}+\overset{k}{\underset{i=1}{\sum }}\beta iX_{i}{}^{2}+\sum^{\;}\overset{.}{\underset{i\leq 1}{\sum }}\beta \mathrm{ij}X_{i}X_{j}+\varepsilon$$

where:

Y = response function (for this study it is the CS),

B0 = constant coefficient,

Bi, Bii, and Bij = factor of the linear, quadratic, and interactive expressions, respectively [4].

In this study, the quadratic model was used for the prediction.

### 2.3. Test and Sample Preparation Method

**Figure 3:**
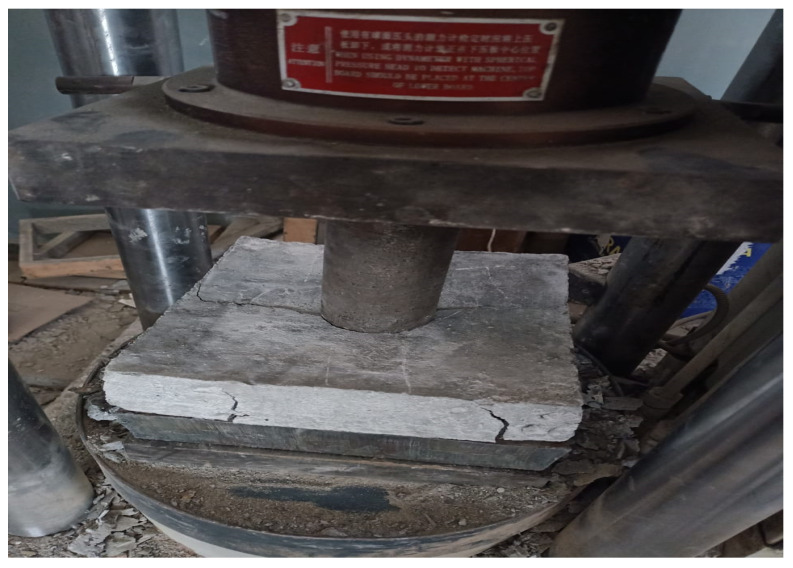
Punching strength setup.

The flow of concrete was determined as per ASTM [37]. The compressive strength was measured using a standard cylinder (150 × 300 mm) in accordance with ASTM standards [38]. Similar cylinders of standard dimensions (150 × 300 mm) were cast and tested for split tensile strength in accordance with ASTM standards [39]. Punching shear was performed on a concrete slab specimen of 500 × 500 mm. For this test, a special kind of assembly was made that represented the punching mechanism in the slabs in concrete structures, as shown in Figure 3. To find out the punching shear strength of the concrete slabs, the slab specimens were removed from the curing procedure and were left to dry in the open air for two hours. Then, a steel baseplate was placed in the compressive testing machine. After that, the slab specimen was placed over the baseplate, and it was made sure that the center of the slab specimen lay at the center of the baseplate. Then, the central point was marked on the top surface of the slab, and the punching rod was placed on the central marked point, as shown in Figure 3. The loading was started by turning the loading switch on and waiting until the rod punched in the slab. The maximum resistive punching load of the slab specimen was noted down.

For each test, three specimens were tested at 7, 28, and 56 days, and the mean value of the specimens was considered the strength of the experiment. The ASTM C-31 [40] method was followed to prepare the specimens, and compaction was done manually by Roding in three layers with 25 blows to each layer. Before the mixing process started, the required quantity of concrete ingredients was weighed by the weighing system. The mixer rate was kept constant at 30 rev/min for the blending of ingredients. Initially, coarse and RCA were put into the drum and then sand. Each ingredient was dry-blended with the essential amount of OPC and WG, water was inserted over time, and blending was performed for around 10 minutes for all batches. A total of fifteen mixed proportions were prepared, i.e., one control mix, six mixes individually with varying percentages of WG and RCA, and eight with combined substitutions of WG and RCA which were determined from statistical analysis. A total of 192 standard sizes were cast and tested as per the specified period of curing. Table 4 depicts the mixed proportion of control and individual mixes.

## 3. Results and Discussion

### 3.1. Workability

**Figure 4:**
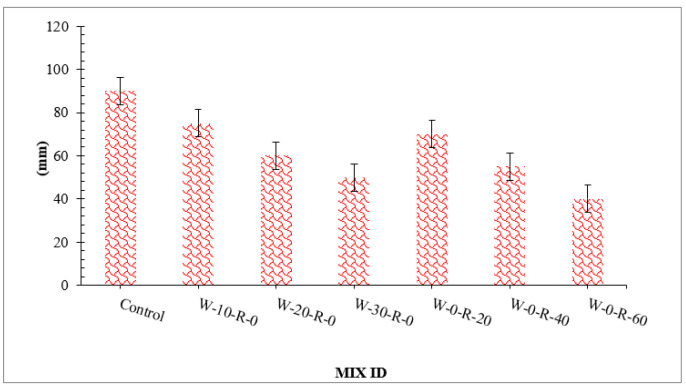
Slump test result.

Workability can be defined as the ease of concrete mixing, transportation, placing, and compacting. Workability directly affects the mechanical performance of concrete. More workable concrete results more compact dense concrete [41]. Figure 4 depicts the slump flow of concrete with different doses of WG. RCA slump value decreased as the percentage of waste glass increased compared to that in the control mix.

The maximum slump was attained at 0% replacement of waste glass, and the minimum slump was achieved at 30% substitution of waste glass. Research also shows that the workability of concrete reduces with the replacement of waste glass [42]. The decrease in workability was because of a larger surface area of waste glass, as shown in Table 2, which required more cement paste to coat them, and hence less cement paste was available for lubrication. Although some studies show that waste glass does not absorb water from the concrete mix, more water is available for lubrication, resulting in more workable concrete [43]. RCA decreased the workability of concrete by absorbing more water from the concrete due to its porous nature and as a result, there was no free water available for lubrication. Research also reports that the workability of concrete decreases with the substitution of RCA due to rough texture, which enhances internal friction between concrete ingredients [42].

### 3.2. Compressive Strength of Concrete

**Figure 5:**
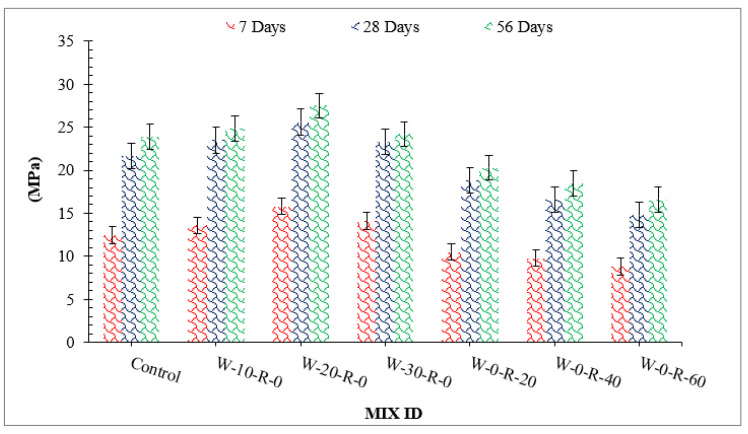
Compressive strength.

Figure 5 shows compressive strength with differing dosages of WG and RCA. Based on work results, compressive strength of concrete improved with substitution of waste glass up 20% and then decreased due to lack of workability.

The pozzolanic reaction of waste glass is responsible for the increased compressive strength. Silica present in waste glass reacts with CH (formed during hydration of cement) and converts it into CSH gel, which provides additional binding property. As a result, strength increases. It can also be observed that improvement in compressive strength at an early stage (seven days) was not significant with the substitution of waste glass, as the pozzolanic reaction proceeds slowly [44]. However, 28 and 56 days of curing considerably improved compressive strength. It is also mentioned that improved compressive strength is due to micro filling of WG, which gives denser concrete [4]. However, a decrease in compressive strength was observed beyond 20% replacement of waste glass owing to a lack of workability, resulting in more voids in hardened concrete. Additionally, a study shows that a higher dose of waste glass (30%) results in less compressive strength due to the dilution effect, which causes an alkali-silica reaction. The amount of calcium hydrates (CH) is consumed by the chemical reaction with silica present in waste glass which gives secondary cementitious material (CSH gel), but at a higher dose of waste glass, might be caused by an alkali-silica reaction due to excessive unreactive silica. As for RCA, compressive strength decreased with substitution of RCA as compared to that of control, with minimum compressive strength at 60% substitution of RCA. The physical nature of RCA causes it to absorb more water, resulting in porous concrete with lower compressive strength. The compressive strength of recycled aggregate concrete has also been observed to be reduced due to unreactive cement [45]. No free water is available for the hydration process since RCA absorbs water from concrete. In addition, lack of workability tends to cause more voids, resulting in lower compressive strength.

**Figure 6:**
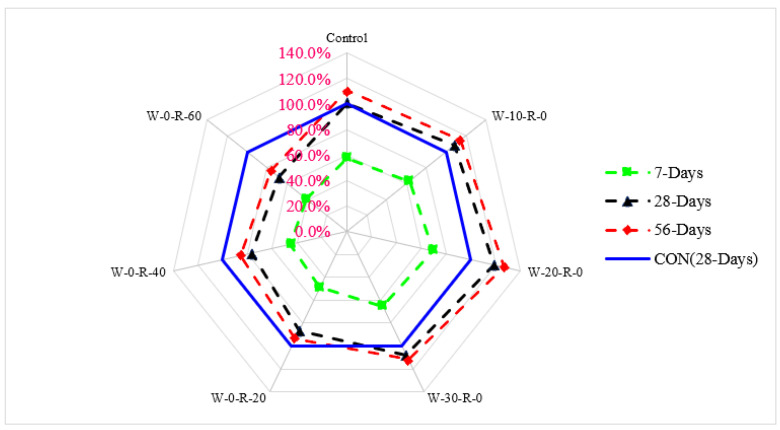
Relative compressive strength.

A relative analysis of compressive strength concerning 28 days of control concrete is shown in Figure 6. Compressive strength at seven days of curing was 31% and 52% lower than that of reference concrete (28 days control concrete) at 20% substitution of waste glass and 20% substitution of RCA, respectively. Compressive strength at 20% substitution of waste glass was 19% higher than that in reference concrete, and at 20% substitution of RCA was 14% lower than that of reference concrete at 28 days of curing. Compressive strength at 56 days of curing was 27% higher than that of reference concrete at 20% substitution of waste glass and only 7.0% lower than that of reference concrete at 30% substitution of RCA, respectively.

**Figure 7:**
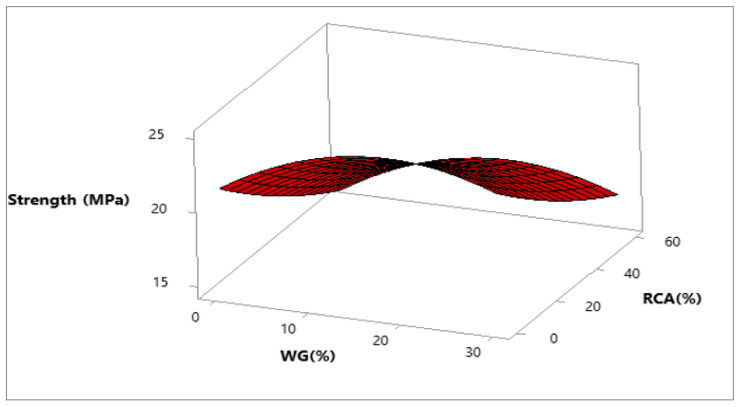
Three-dimensional response surface of compressive strength.

**Figure 8:**
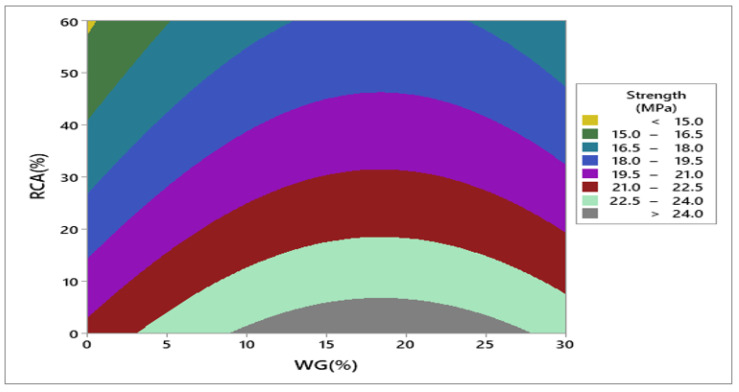
Contour plot of compressive strength.

**Figure 9:**
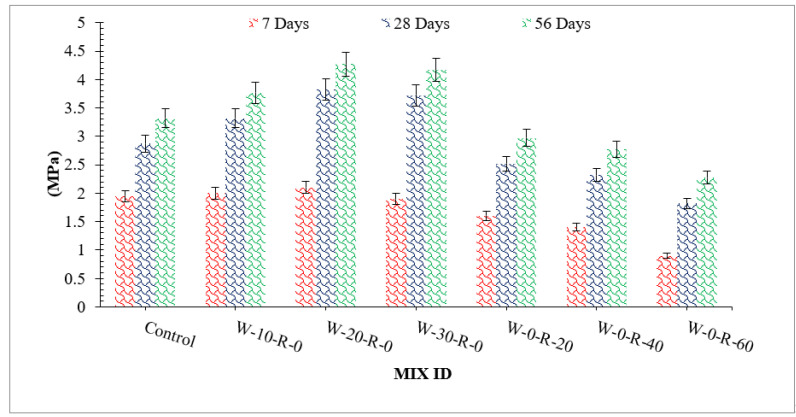
Split tensile strength.

Response surface and contour plot for compressive strength after 28 days of curing are shown in Figure 7 and Figure 8, respectively. It can be observed from the contour plot that RCA decreased compressive strength while WG improved compressive strength. However, concrete made with 20% WG and 30% RCA showed compressive strength (20.5 MPa) approximately equal to the compressive strength of control (21.65 MPa), as shown in Figure 9. Furthermore, concrete with 15% RCA and 20% WG showed a compressive strength of 23 MPa, which was 7.0% higher than the compressive strength of control. Similar doses (15% RCA and 20% WG) were also cast and tested in the laboratory. It can be observed that experimental compressive and predicted compressive strength from the contour plot were approximately equal, which validated the predicted results. Therefore, the overall discussion suggests that RCA up to 40% with a combination of 20% WG could be safely used in cement concrete production without adverse effects on compressive strength.

### 3.3. Split Tensile Strength of Concrete

**Figure 10:**
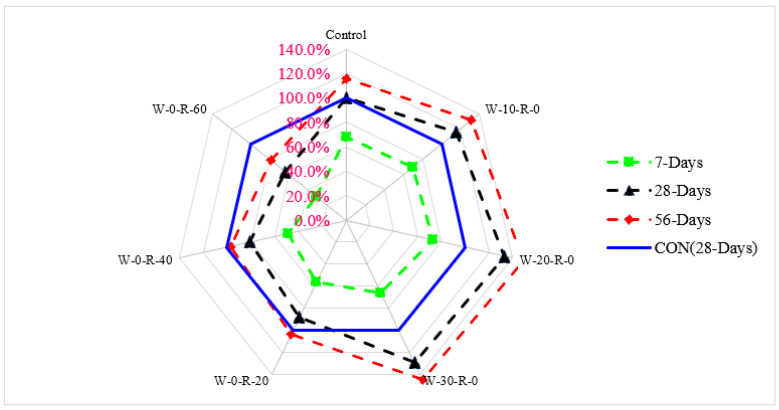
Relative split tensile strength.

Figure 9 shows the split tensile strength of concrete with different dosages of concrete, and relative analysis of split tensile strength with respect to reference concrete is shown in Figure 10. Split tensile strength is a function of compressive strength. Split tensile strength is about 10–15% of compressive strength. Similar to compressive strength, split tensile strength increased up to 20% replacement and then decreased. Additionally, a study concluded that split tensile strength of concrete is improved up to 20% substitution of waste glass and then decreases due to lack of workability [42]. At 20% waste glass replacement, the maximum split tensile strength was achieved, which was over 48% greater than that of reference concrete after 56 days of curing. The glass had a positive impact because of a pozzolanic reaction that provided extra binding properties, resulting in denser concrete. Micro filling of waste glass resulted in denser concrete, which led to more split tensile strength. It could also be observed that waste glass improved split tensile strength more effectively than compressive strength. A study also shows that waste glass improves split tensile strength more effectively than compressive strength due to the improvement of cement paste strength [42]. It has also been reported that concrete has less split tensile strength due to lower cement paste strength [4]. Substitution with waste glass improved the split tensile strength of concrete due to the formation of secondary CSH, which increased the binding property of cement paste. As for RCA, split tensile strength decreased with the substitution with RCA due to its rough surface texture and porous nature. Maximum split tensile strength was achieved at 0% substitution of RCA, and minimum split tensile strength was achieved at 60% substitution of RCA. That was because of RCA’s physical properties, which allowed it to absorb more water, resulting in porous concrete with lower split tensile strength. A study also observed that the split tensile strength of concrete decreases with the substitution of RCA [9].

**Figure 11:**
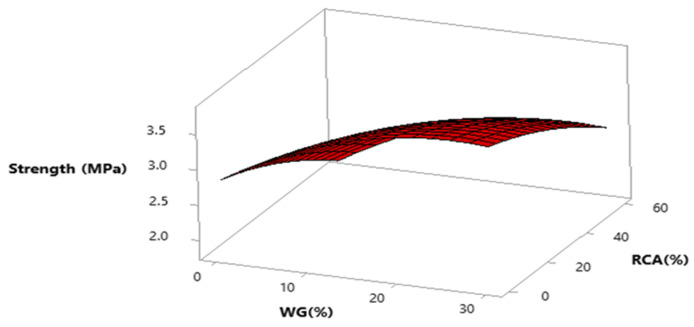
Three-dimensional response surface of split tensile strength.

**Figure 12:**
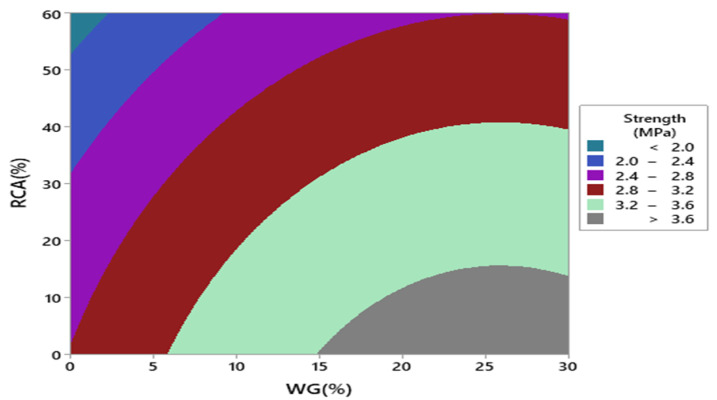
Contour plot of split tensile strength.

Response surface and contour plot for split tensile strength after 28 days of curing are shown in Figure 11 and Figure 12, respectively. It can be observed from the contour plot that RCA decreased split tensile strength while WG improved split tensile strength. Contour plots were used to optimize WG and RCA for split tensile strength. It can be observed that concrete made with 20% WG and 30% RCA showed split tensile strength (3.0 MPa) that was approximately equal to the split tensile strength of control (2.87 MPa). Similar doses of (20% WG and 40% RCA) were also cast and tested in the laboratory after 28 days of curing. It could be observed that experimental and predicted split tensile strength from the contour plot were approximately equal, which validated the predicted split tensile strength.

**Figure 13:**
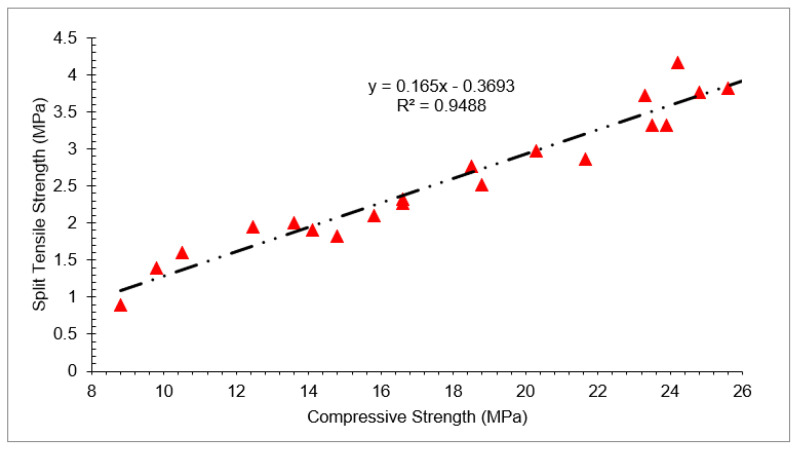
Correlation between compressive and split tensile strength (7, 28 and 56 days).

The correlation between compressive strength and split tensile strength with varying doses of waste glass and RCA at different days of curing is shown in Figure 13. A strong correlation existed between compressive strength and split tensile strength with an R^2^ value greater than 90%.

The following equation was developed based on experimental tests with different doses of RCA and WG.

f_sp_ = 0.165 × f_c_ − 0.36 (3)

where

f_sp_ = split tensile strength_,_ f_c_ = compressive strength

**Figure 14:**
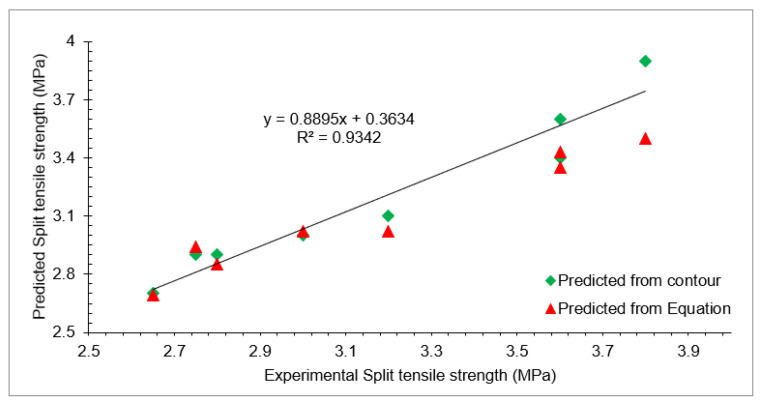
Correlation between experimental and predicted split tensile strength.

Experimental split tensile strength predicted from the contour plot and predicted from Equation (3) with different doses of waste glass and RCA at 28 days of curing is shown in Table 5. Furthermore, a regression model for experimental split tensile strength, predicted from contour plot and Equation (3) with different doses of waste glass and RCA at 28 days of curing is shown in Figure 14. A strong co-relation existed among experimental split tensile strength predicted from contour plot and Equation (3), with an R^2^ value greater than 90%.

### 3.4. Stress-Strain Curve

**Figure 15:**
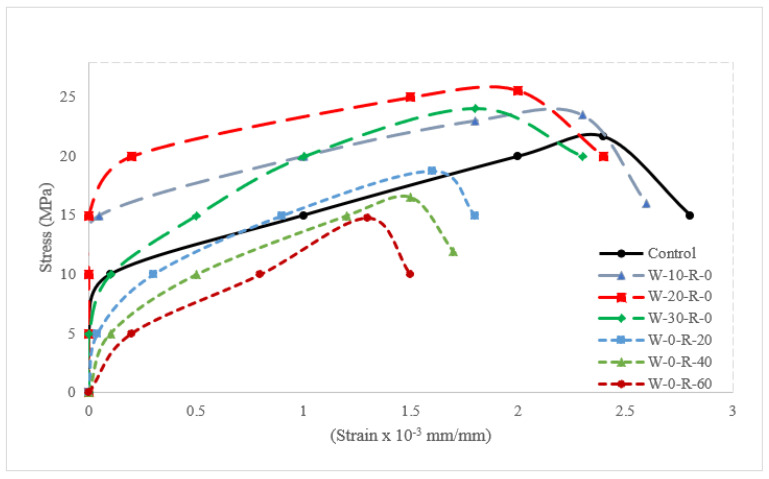
Stress-strain curve of various doses of waste glass and RCA.

Figure 15 depicts the stress-strain curve of various waste glass and RCA dosages. The stress-strain curves for waste glass and RCA both have rising and falling sections, similar to those of conventional concrete. The tension required to induce the first strain of the waste glass was larger than that in reference concrete according to the test results. It was due to the pozzolanic reaction which gave the secondary cementitious compound (CSH). CSH increased the binding properties of cement paste, hence more stress was required to initiate strain. Additionally, the stress required to initiate the initial strain increased due to the micro filling of waste glass, which gave denser concrete. However, in the case of RCA, the stress required to initiate the initial strain was lower than that of reference concrete. It was due to the physical nature of RCA (voids), which absorbed more water from the cement paste and hence some part of cement remained unreactive and formed weak pockets. Similarly, due to the combined pozzolanic reaction and micro filling of waste glass, the ultimate stress increased with substitution of waste glass up to 20% and decreased due to the lack of workability. In the case of RCA, the ultimate stress decreased with the substitution of RCA. Although waste glass increased ultimate stress considerably, ultimate strain decreased with the substitution of waste, which resulted in more rigid concrete (brittle failure), which is not acceptable for any structural member. Therefore, it is recommended to use some tensile reinforcement (fibers) to improve the tensile capacity of concrete (ductile failure). In the descending portion of the stress-strain curve, reference concrete and concrete made with waste were approximately the same. However, in concrete made with RCA, the descending portion of the stress-strain curve was steeper.

### 3.5. Punching Strength of Concrete

**Figure 16:**
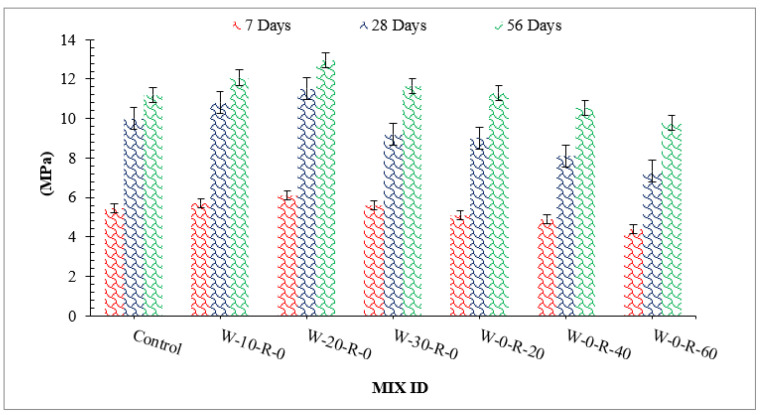
Punching strength.

Figure 16 shows the punching strength with varying dosages of WG and RCA. Similar to compressive strength, the punching strength of concrete improved with the substitution of waste glass up 20% and then decreased due to lack of workability. Maximum punching strength was observed at 20% substitution of waste glass, and minimum punching strength was observed at 0% substitution of waste glass. The pozzolanic reaction of waste glass, which gives secondary cementitious material and micro filling, resulted in a more compact mass that positively influenced the punching strength of concrete. However, a decrease in punching strength was observed beyond 20% replacement of waste glass due to lack of workability, which resulted in more voids in hardened concrete. Additionally, a study shows that a higher dose of waste glass (30%) results in less strength due to the dilution effect, which causes an alkali-silica reaction [42]. The amount of calcium hydrates (CH) is consumed by a chemical reaction with silica present in waste glass, which gives secondary cementitious material (CSH gel), but at a higher dose of waste glass might be caused by an alkali-silica reaction due excessive unreactive silica. As for RCA, punching strength decreased with substitution of RCA as compared to that of control, having minimum punching strength at 60% substitution of RCA. The physical nature of RCA caused it to absorb more water, resulting in porous concrete with lower punching strength. The strength of recycled aggregate concrete has also been observed to have decreased due to unreactive cement [45]. RCA absorbs water from concrete, leaving no water for the hydration process. In addition, lack of workability tends to cause more voids in hardened concrete, leading to lower punching strength.

**Figure 17:**
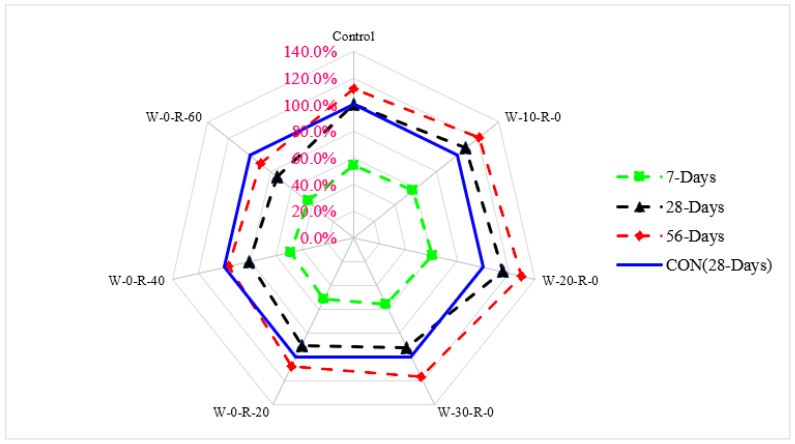
Relative punching strength.

A relative analysis of punching strength concerning 28 days of control concrete punching strength is shown n in Figure 17. Punching strength at 7 days of curing was 29% and 49% lower than that of reference concrete (28 days control concrete) at 20% substitution of waste glass and 30% substitution of RCA, respectively. Punching strength at 20% substitution of waste glass was 15% higher than that of reference concrete and at 20% substitution of RCA was 10% lower than that of reference concrete at 28 days of curing. Punching strength at 56 days of curing was 29% higher than that of reference concrete and at 20% substitution of RCA was approximately equal to that of reference concrete.

**Figure 18:**
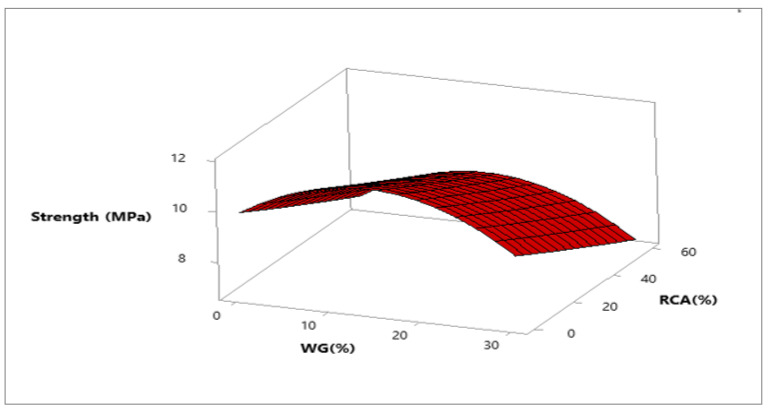
Three-dimensional response surface of punching strength.

**Figure 19:**
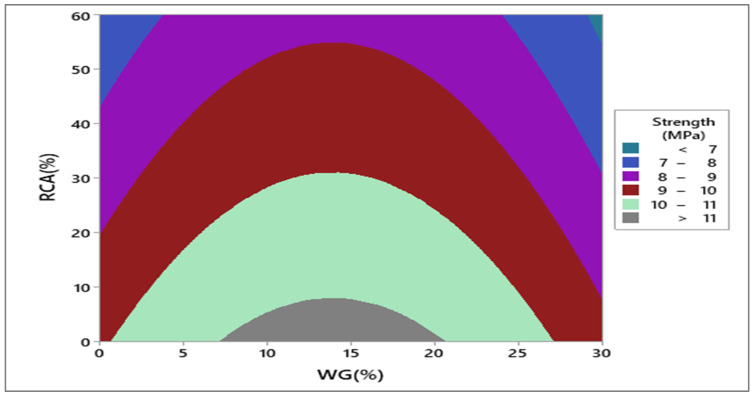
Contour plot for punching strength.

Response surface and contour plot for punching strength after 28 days of curing are shown in Figure 18 and Figure 19, respectively. It can be observed from the contour plot that RCA decreased punching strength while WG increased punching strength.

However, concrete made with 20% WG and 40% RCA showed punching strength (9.1 MPa) that was approximately equal to the punching strength of control (10 MPa), as shown in Figure 19. Similar doses (40% RCA and 20% WG) were also cast and tested in the laboratory. It could be observed that experimental punching strength and predicted punching strength from the contour plot were approximately equal, which validated the predicted results.

**Figure 20:**
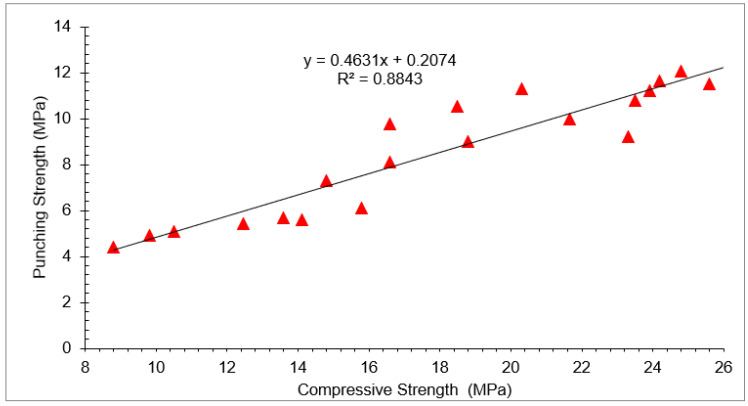
Correlation between compressive and punching strength (7, 28, and 56 Days).

Correlation between compressive strength and punching strength with varying doses of waste glass and RCA at different days of curing is shown in Figure 20. A strong correlation existed between compressive strength and punching strength with an R^2^ value approximately equal to 90% (88%). The following equation was developed based on experimental tests with different doses of RCA and WG.

f_pu_ = 0.463 × fc − 0.207 (4)

where

f_pu_ = punching strength_,_ f_c_ = compressive strength

**Figure 21:**
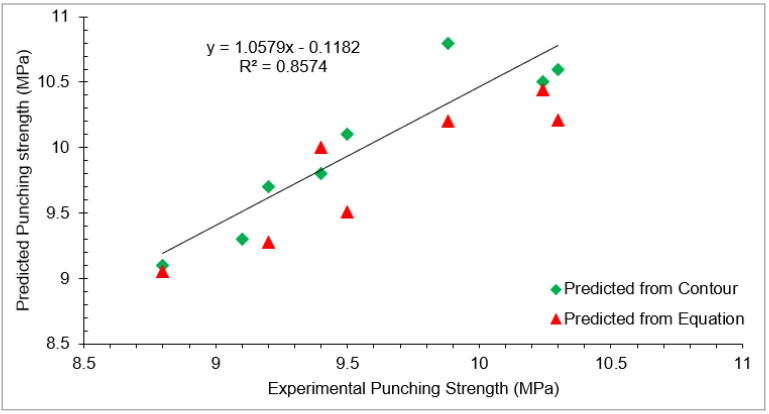
Correlation between experimental and predicted punching strength.

Experimental punching strength and punching strength predicted from the contour plot and Equation (4) with different doses of waste glass and RCA at 28 days of curing are shown in Table 6. Furthermore, a regression model for experimental punching strength, punching strength predicted from the contour plot, and punching strength predicted from Equation (4) with different doses of waste glass and RCA at 28 days of curing is shown in Figure 21. A strong co-relation existed among experimental punching strength, punching strength predicted from the contour plot, and punching strength predicted from Equation (4), with an R^2^ value approximately equal to 90%.

## 4. Conclusions

In this research, a step towards sustainable concrete was made by incorporating WG and RCA. The substitution ratios of WG were 0%, 10%, 20%, and 30% by weight of cement, and those of RCA were 0%, 20%, 40%, and 60% by weight of coarse aggregate. A detailed conclusion based on test results is given below.

- As the percentage of WG and RCA increased, the workability of concrete decreased. It was related to the physical characteristics of RCA and WG, which had a larger surface area.
- WG did not give a significant improvement in strength in the early days (seven days), as the pozzolanic reactions proceeded slowly. However, a considerable improvement in strength was observed at 28 and 56 days of curing.
- Maximum strength was obtained at 20% substitution of WG. At 28 days of curing, compressive strength was 27% higher than that of reference concrete, while punching strength was 29% higher than that of reference concrete.
- RCA lowered the mechanical performance of concrete by absorbing more water from it, leaving less or no water available for hydration and workability.
- A successful statistical analysis (response surface methodology) predicted various mechanical properties and optimized WG and RCA. The optimal doses of WG and RCA (20% WG and 30% RCA) were predicted from the statistical analysis, which showed compressive strength approximately equal to that of the reference concrete. Furthermore, the same doses of WG and RCA (20% WG and 30% RCA) were cast and tested experimentally. It could be observed that the predicted and experimental values were comparable, which validated the predicted results.

Finally, the overall study demonstrated that RCA could be successfully utilized with combined substitution of WG without any negative effect on the mechanical performance of concrete. However, it could be observed that combined substitution of WG and RCA up to some extent improved the mechanical performance of concrete. However, concrete is still weak in tension, which results in abrupt failure. Therefore, further research is recommended with addition of steel fibers or natural fibers (coconut fibers, etc.) to improve the tensile capacity of concrete.

## Figures and Tables

**Table 1:** Properties of Ordinary Portland Cement (OPC).

Chemical Name	Percentage (%)	Physical Name	Results
CaO	63.7	Size	Less than 75 µm
SiO_2_	22.9	Fineness	97%
Al_2_O_3_	4.2	Normal Consistency	29%
Fe_2_O_3_	3.5	Initial Setting Time	38 min
MgO	3.0	Final Setting Time	430 min
SO_3_	1.4	Specific surface	320 m^2^/kg
K_2_O	0.5	Soundness	1.60%
Na_2_O	0.8	28 days compressive Strength	42 MPa

**Table 2:** Properties of waste glass.

Chemical Name	Percentage (%)	Property Name	Value	Mineralogy	Quartz
CaO	60.7	Size	Less than 75 µm	Loss of ignition (%)	4.8
SiO_2_	24.9	Fineness	98%	Water content (%)	4.1
Al_2_O_3_	4.7	Specific surface	342 m^2^/kg	Clay (%)	6.1
Fe_2_O_3_	2.0	Specific gravity	3.1	TOC (mg/kg)	64.9
MgO	1.9	Absorption	0.01	Hydrocarbons (mg/kg)	75.13
SO_3_	2.5			Sulphates (%)	3.08
K_2_O	0.2			HHV (kJ/kg)	170.5
Na_2_O	1.1			PCB (mg/kg)	0.28

**Table 3:** Fine and coarse aggregate properties.

Property	Fine Aggregate	Coarse Aggregate	RCA
Particle Size	4.75 to 0.075 mm	25 to 4.75 mm	25 to 4.75 mm
Fineness Modulus	2.53	3.8	2.2
Absorption Capacity	4.26%	2.28%	3.3%
Moisture Content	2.8%	0.55%	0.75%
Specific Gravity	2.91	2.45	2.35

**Table 4:** Quantification of materials per cubic meter.

Mix ID	Cement (kg)	Fine Aggregate (kg)	Coarse Aggregate (kg)	WG (kg)	RCA (kg)	Water (kg)	Admixture (kg)
Control	385	550	1150	-	-	180	4.25
W-10-R-0	346.5	550	1150	38.5	-	180	4.25
W-20-R-0	308	550	1150	77	-	180	4.25
W-30-R-0	265.5	550	1150	115.5	-	180	4.25
W-0-R-20	385	550	920	-	230	180	4.25
W-0-R-40	385	550	690	-	460	180	4.25
W-0-R-60	385	550	460	-	690	180	4.25

**Table 5:** Experimental and predicted split tensile strength.

WG%, RCA%	Compressive Strength from Contour Plot	Experimental Split Tensile Strength	Split Tensile Strength from Contour Plot	Split Tensile Strength from Equation (3)
10, 10	23.0	3.60	3.40	3.43
10, 20	20.5	3.20	3.10	3.02
10, 30	19.5	2.80	2.90	2.85
10, 40	18.5	2.65	2.7	2.69
20, 10	22.5	3.80	3.9	3.35
20, 20	21.0	3.60	3.5	3.10
20, 30	20.5	3.00	3.0	3.02
20, 40	20.0	2.75	2.9	2.94

**Table 6:** Experimental and predicted punching strength.

WG%, RCA%	Compressive Strength from Contour Plot	Experimental Punching Strength	Punching Strength from Contour Plot	Experimental Punching Strength from Equation (4)
10, 10	23.0	10.24	10.5	10.44
10, 20	20.5	9.88	10.8	9.28
10, 30	19.5	9.4	9.8	8.8
10, 40	18.5	9.1	9.3	8.37
20, 10	22.5	10.3	10.6	10.21
20, 20	21.0	9.5	10.1	9.51
20, 30	20.5	9.2	9.7	9.28
20, 40	20.0	8.8	9.1	9.05

## Data Availability

All Data are available in the manuscript.
